# Phycian assisted suicide: A Swiss perspective - a liberal view

**DOI:** 10.1192/j.eurpsy.2024.119

**Published:** 2024-08-27

**Authors:** G. Stoppe

**Affiliations:** MentAge, Basel, Switzerland

## Abstract

**Abstract:**

Switzerland is a country in which the liberal tradition is cultivated and every citizen’s free decision is honoured. Associations such as EXIT or Dignitas, which advocate the right to self-determined death, were formed here early on. They see themselves as completing the Age of Enlightenment, where the end result is an individually self-determined death. The Swiss federal government is therefore reluctant to define criminal offences. However, it is regulated that active euthanasia is prohibited. The organisations mentioned are also not allowed to act for their own benefit.

Meanwhile, the number of assisted suicides is higher than the number of suicides. The extent to which the one phenomenon is related to the other is open to debate.

The topic is controversial among the general public and the medical profession. Nevertheless, the Swiss Academy of Medical Science has published guidelines on dealing with dying and death, which also deal with physician-assisted suicide.

Various associations, including the umbrella organisation for suicide prevention in Switzerland, Ipsilon, are in favour of special protection for vulnerable groups. Some of them also recommend that civil law provisions be made for the process of assisted suicide.

**Disclosure of Interest:**

None Declared

